# The Origin of Behavioral Bursts in Decision-Making Circuitry

**DOI:** 10.1371/journal.pcbi.1002075

**Published:** 2011-06-23

**Authors:** Amanda Sorribes, Beatriz G. Armendariz, Diego Lopez-Pigozzi, Cristina Murga, Gonzalo G. de Polavieja

**Affiliations:** 1 Instituto Cajal, Consejo Superior de Investigaciones Científicas, Madrid, Spain; 2 Department of Theoretical Physics and Instituto “Nicolás Cabrera” de Física de Materiales, Universidad Autónoma de Madrid, Madrid, Spain; 3 Departamento de Biología Molecular and Centro de Biología Molecular Severo Ochoa (CSIC-UAM), Instituto de Investigación Sanitaria Princesa, Universidad Autónoma de Madrid, Madrid, Spain; Indiana University, United States of America

## Abstract

From ants to humans, the timing of many animal behaviors comes in bursts of activity separated by long periods of inactivity. Recently, mathematical modeling has shown that simple algorithms of priority-driven behavioral choice can result in bursty behavior. To experimentally test this link between decision-making circuitry and bursty dynamics, we have turned to *Drosophila melanogaster*. We have found that the statistics of intervals between activity periods in endogenous activity-rest switches of wild-type *Drosophila* are very well described by the Weibull distribution, a common distribution of bursty dynamics in complex systems. The bursty dynamics of wild-type *Drosophila* walking activity are shown to be determined by this inter-event distribution alone and not by memory effects, thus resembling human dynamics. Further, using mutant flies that disrupt dopaminergic signaling or the mushroom body, circuitry implicated in decision-making, we show that the degree of behavioral burstiness can be modified. These results are thus consistent with the proposed link between decision-making circuitry and bursty dynamics, and highlight the importance of using simple experimental systems to test general theoretical models of behavior. The findings further suggest that analysis of bursts could prove useful for the study and evaluation of decision-making circuitry.

## Author Summary

It has long been observed that animal movement tends to come in bursts of activity. This has been seen in many animal species, ranging from small insects to even human activity patterns. The underlying mechanisms remain unknown, but recently a mathematical model showed that it could be due to priority-driven choice behavior. If the animals decide what to do next depending on the relative priorities of the choices at hand, the behavior becomes bursty, while if the animals simply act directly on cues coming from the outside, their behavior becomes less structured and more random. To test if decision-making processes affect behavior in bursts, we studied the fruit fly (*Drosophila melanogaster*), because of the powerful genetic tools available. We manipulated a part of the brain known as the mushroom body, and neurons that form the dopaminergic system, since both had previously been found to disrupt normal choice behavior in the fly. In particular we found that high levels of dopamine made the flies' activity pattern less structured, and that parts of the mushroom body circuitry also affected burstiness. Our findings are thus consistent with the idea that decision-making processes could be important for understanding animal and human activity patterns.

## Introduction

Bursts in behavior are common [1]–[10], but only recently have there been modeling efforts to understand its origin from a behavioral point of view [11]–[14]. The queued priority list model was first proposed by Barabási [11], [12] to explain why inter-event times in human activities such as e-mail and letter writing have a bursty nature. In the proposed model behavioral bursts are the consequence of an internal decision-making process, with tasks generally being executed in order of perceived relative priority and an additional random component. Later work has argued that the distributions in [11] might not follow a power law but the log-normal distribution [15], or that e-mail communication data can instead be explained with a cascading nonhomogeneous Poisson process [14] or as a sum of Poisson processes [16]. Regardless of the particular model implementations or resulting distributions, however, an important ingredient of the Barabási model is the proposed relationship between decision-making and behavioral bursts.

The link proposed by the Barabási model between behavioral bursts and an underlying decision-making algorithm is not a logical necessity. Indeed, alternative mechanisms proposed to explain behavioral bursts have been based on random processes, for example by a cascading nonhomogeneous Poisson process [14] or by a sum of Poisson processes with different mean rates [16]. The link between behavioral burstiness and decision-making thus needs experimental validation. We therefore set out to experimentally determine whether neuronal circuitry necessary for decision-making is also necessary for the bursts seen in behavior.

We found *Drosophila melanogaster* an ideally suited model system for this test. *Drosophila* has been shown to have a complex decision-making behavior and not simply hard-wired stimulus-responses [17]–[25]. Flies can initiate behavior [17], [21], probabilistically activate a given action from a range of possible ones and learn to use the particular actions that give the target result [17], [19], [23], [24]. Decisive components of the fly decision-making circuitry have been identified and characterized [18], [20], [22]–[25]. In particular, dopaminergic neurons have been found to be necessary for decision-making in tethered flight [20] and in olfactory-driven [24] and visually-driven choices of walking flies [25]. Dopaminergic neurons have also been found to form a reinforcement circuit establishing which actions are appropriate [24]. The neuroanatomical substructure known as the mushroom body (MB), long known for its implication in olfactory memory formation and retrieval [e.g. reviewed in 26] has also been found to be necessary for decision-making in tethered flight [18], [20] and implicated in visual attention-like behavior [27].

The complex decision-making behavior of *Drosophila* already shows components consistent with a priority-based model like that of Barabási. For example, the activation of actions is probabilistic [17], [19], [23], [24] and its brain has components that can reinforce some actions, that is, to give them higher priority [24]. Interestingly, *Drosophila* has also been shown to have intrinsic behavioral variability. This has been found to be influenced by the ellipsoid-body (EB) [28], [29], a substructure of the central complex (CX), which has also been implicated in visuo-motor control [30] and visual memory tasks [31]. Notably, the MB has also been implicated in behavioral variability. Concretely, it has been argued to be a site that establishes a balance between the variability needed for flexibility and the inflexibility of habit formation [23].

Powerful genetic tools for targeted neuronal silencing have been developed for *Drosophila* [32], [33]. To test the link between decision-making and burstiness in *Drosophila*, we selectively silenced parts of the MB, or modified dopaminergic signaling, components previously found to disrupt decision-making [20], and found that the flies' inherent burstiness changed, a result thus consistent with the core idea of the Barabási model [11], [12].

To study burstiness in *Drosophila*, we measured the spontaneous walking activity of flies with the DAM2 System (Trikinetics, MA), which is a detector system with infra-red beams that cross through the center of 32 tubes, each one containing a single fly. When a fly crosses the beam an activity event is registered for that fly. Data were sampled in 1 minute bins, and separated into activity bouts (ABs) and inter-activity intervals (IAIs) for analysis (see Activity Assay).

## Results

### Burstiness in *Drosophila* is described by a Weibull distribution

A hallmark of bursty dynamics is that the time intervals between events follow non-Poissonian statistics, with long and short time intervals being more common than in the random (Poissonian) case. In cases like these, calculating the mean event duration or mean inter-event interval duration offers poor descriptions of the underlying behavior. Instead, an alternative approach is to fit an analytical function to the empirical statistical distribution. Three common problems with this approach have however been noted. First, it is common to fit the data to a power law [1], [2], [4]–[7], [9]–[12], [21], [28], [29], [34], but this procedure can be problematic [35], [36]. Usually, the experimental distribution is given in a log-log plot and it is then fitted to a straight line for a range of the data. However, log-log plots often give the impression of linear trends for part of the data, and thus spurious results can be obtained [35], [36]. Second, it has been noted that apparent bursty behavior can emerge as a consequence of pooling from a population of Poisson individuals with different Poisson rates [16]. Third, this can also happen as a consequence of circadian rhythms in the activity patterns [14]. We analyzed our data with the aim to avoiding these three problems. First, we found that the Weibull distribution has an excellent fit to the entire range of inter-activity intervals (IAIs) and not simply to a particular region. Second, we obtained data for individual flies and show that each follows a Weibull distribution. Third, we analyzed separately day (lights on) and night (lights off) data instead of taking the IAIs for an entire day, thus avoiding a possible circadian rhythm influence. Since *Drosophila* day-time activity is usually non-stationary with a mid-day ‘siesta’, we have focused this study on the more stationary night period, **Figure S1A**.

**Figure 1:**
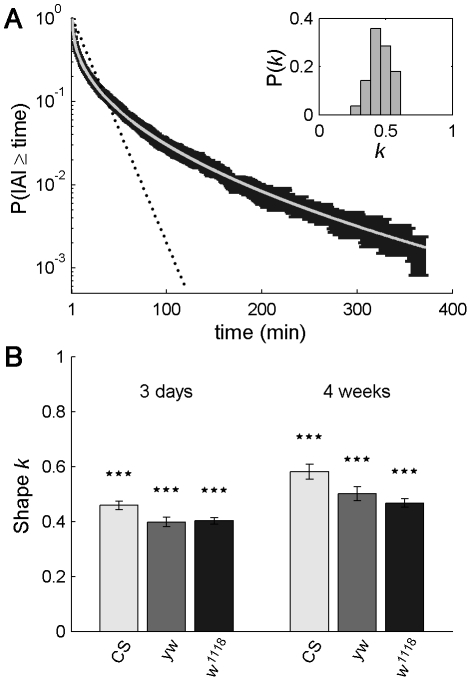
*Drosophila* inter-activity intervals (IAIs) follow the Weibull distribution. (A) Mean IAI survival distribution of 28 *Canton-S* flies during the dark period (black, mean ± standard error) shows a clear deviation from the exponential distribution corresponding to the IAIs of a Poisson process (dotted line, same mean as actual IAI distribution). The Weibull distribution (light grey line) fits data accurately (r^2^ = 0.998), with *k* = 0.45, *λ* = 6.0. Individual flies also have IAIs following the Weibull distribution. Inset: Distribution of *k* values obtained for the same data set but performing individual fits (mean fit r^2^ = 0.92±0.07 s.e.m.). Each fly shows bursty dynamics with *k* = 0.46±0.08 s.e.m. (B) Shape parameter *k* for young (3 days, left) and adult (4 weeks, right) *Canton-S* (*CS*) flies, *yellow-white* (*yw*) and *w^1118^* flies. All show bursty dynamics, with *k*<1, significantly different from the Poissonian *k* = 1 case with *p*<10^−7^. Day and night data are treated separately as the activity dynamics are different; in Figure S1A we present the corresponding daily activity patterns for the 3-day-old *CS*, *yw* and *w^1118^*. Number of flies n = 28–32.

The mean complementary cumulative (survival) distribution of IAIs for 3-day-old flies with the standard genetic background *Canton-S* (CS) (**Figure 1A**, black error bars) showed a clear deviation from Poissonian behavior (**Figure 1A**, dotted line for Poisson distribution with the same mean IAI as data). Data correspond with flies displaying bursty dynamics, with many periods of high activity separated by long periods of inactivity. We found that the complementary cumulative Weibull distribution,
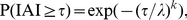
(1)fitted very well the experimental IAI complementary cumulative distribution for all the range of inter-activity intervals (**Figure 1A**, light grey line, r^2^ = 0.998, n = 28; see Burstiness Analysis and **Figure S2** for fitting technique). The initial portion of the empirical IAI distribution can be fitted to a line in a log-log plot and that has been used to argue in favor of a power law ([2], [4], [29] & **Figure S1B**), but many distributions appear straight in a log-log plot for part of the data [35], [36] and this kind of plots are not accurate enough to find the underlying exponent [36, and references therein]. More importantly, the Weibull distribution fits the data for the entire experimental interval of IAI values. This means that the tail of the distribution is heavy but less so than with a power law, and also that there is a natural scale. Indeed, the two parameters that characterize the Weibull distribution are the scale, *λ* = 6.0, and the shape, *k* = 0.45, **Figure 1A**. The scale parameter is linearly related to the mean IAI (see Supporting Information, Text S1). Importantly for our analysis, the shape parameter allows a parameterization of the degree of burstiness, with *k* = 1 corresponding to the Poisson case and *k*<1 to bursty behavior, burstier the lower its value. The experimental Weibull distribution was not due to a population effect, as each fly is well fitted by a Weibull distribution, with r^2^ = 0.97 (mean)
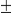
0.02 (s.d.). All flies showed bursty dynamics with *k* = 0.46 (mean)
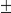
0.08 (s.d.), **Figure 1A** inset. Even more relevant than having a good fit to the data, is the possibility to correctly estimate the underlying parameters that emerge by using the Weibull distribution. We tested with artificial data that our fitting technique correctly extracted the parameters *λ* and *k* for data sizes comparable with the experimental ones (see Burstiness Analysis and **Figure S2**).

In addition to *Canton-S*, we tested two other common genetic backgrounds, *yellow-white* (*yw*) and *w^1118^*, and found that they all had bursty dynamics, **Figure 1B**. Further, we observed that both young (3-day-old) and adult (4-week-old) flies showed bursty dynamics. However, a general decrease in burstiness is observed with aging, as illustrated by a 22.2% mean increase of the shape parameter *k*, **Figure 1B**.

### Burstiness in walking *Drosophila* is mainly due to the inter-event distribution and not to memory effects

**Figure 2:**
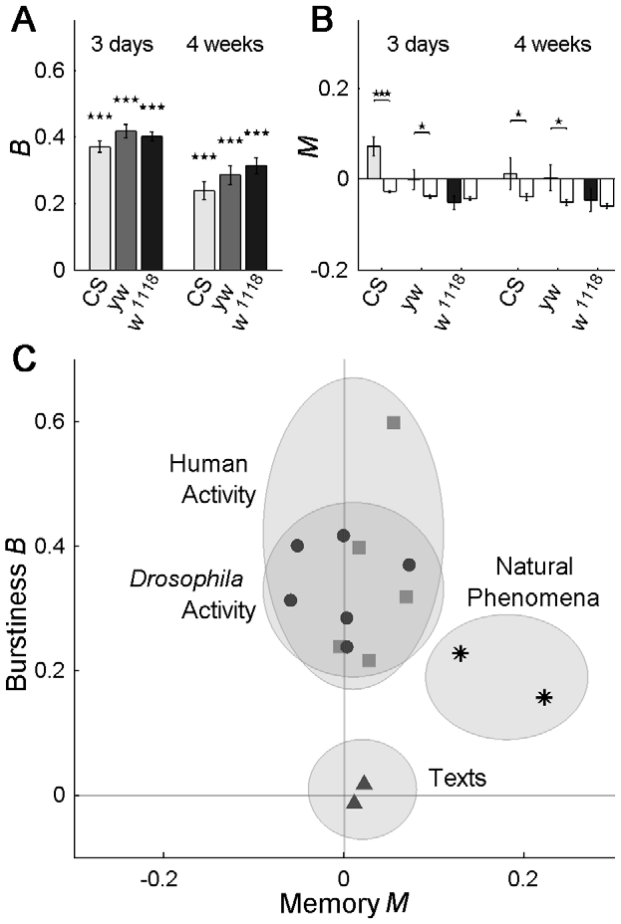
*Drosophila* burstiness is mainly due to the IAI distribution, and not to memory effects. (A) Burstiness parameter *B* for young (3 days, left) and adult (4 weeks, right) *Canton-S* (*CS*), *yellow-white* (*yw*) and *w^1118^* flies, show bursty dynamics with *B*>0 (cf. Figure 1B), significantly different from the Poissonian *B* = 0 case with *p*<10^−7^. Older flies show a decrease of burstiness as compared to younger flies. (B) Burstiness of *Drosophila* IAIs has a small memory component. Significance levels are computed by comparison of actual and shuffled data (white bars). For the genetic background used in this study, *w^1118^*, there is no significant memory. Similar results are found for long-term memory, Figure S4. (C) Burstiness *B* and memory *M* are two different and independent burst-generating mechanisms, here represented in a plane and compared with data for human behavior dynamics, environmental phenomena and texts, taken from [8]. *Drosophila* dynamics fall in the same region as human dynamics, a region clearly separated from both environmental phenomena and texts. Figure S3 gives an overview of the total inter-activity intervals, shape and burstiness, as well as the corresponding values for total activity bout time, shape and burstiness. The same *Drosophila* data were used for Figures 1 and 2, number of flies n = 28–32. Error bars represent mean ± s.e.m.

Different animals, or even the same animal in different times or states, can have stochastic bursts following different statistics. To be able to compare individuals in situations where the distribution can be different, it is convenient to also have a measure of burstiness independent of the distribution. For this, we have used the burstiness parameter *B* [8],
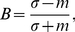
(2)where σ and *m* are the standard deviation and the mean of the IAIs, respectively. The burstiness parameter has values in the range (−1,1), where *B* = 1 corresponds to completely bursty dynamics, *B* = 0 to random event times (Poissonian) and *B* = −1 to periodic activity. The burstiness parameter, when applied to the Weibull distribution, depends only on the shape parameter *k* (i.e., is independent of *λ*) and decreases with *k* (see Supporting Information, Text S1). Walking *Drosophila* display bursty behavior with mean values of *B* in the range of 0.23–0.46, **Figure 2A**. In general we find a strong agreement between the two measures of burstiness, but for finite data the shape parameter *k* is more sensitive to data located on the tail range of the time distribution, while *B* is more dominated than *k* by short time events. The same type of analysis can be applied to the duration of activity bouts (AB), where we also find non-Poisson dynamics, with strong agreement between the two burstiness parameters *k* and *B*, **Figure S3**.

Another source of bursty dynamics, apart from the IAI distribution, are memory effects in the time-series of events [8]. Two systems can have the same IAI distribution, but the system with the stronger memory (i.e. short/long intervals followed by short/long intervals) displays burstier dynamics. We characterized the memory effect *M* with an estimator of the correlation coefficient of consecutive IAIs [8],
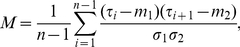
(3)where *n* is the number of IAIs, *m*_1_ (*m*_2_) and σ_1_ (σ_2_) are the mean and standard deviation of the IAIs τ_i_'s (τ_i+1_'s), respectively, with *i* = 1,…,*n*−1. The bursty dynamics found in the three common background strains exhibit mean memory effects in the range [−0.05 0.07], **Figure 2B**, small compared to the values of approximately 0.15–0.2 of other bursty phenomena [8]. Importantly, for *w^1118^*, the genetic background used in the transgenic experiments in this study, there is no significant memory, p>0.5, when comparing actual data against shuffled (memory-less) versions, **Figure 2B**. We also tested for long-range memory effects in the activity. For that, we used detrended fluctuation analysis (DFA) [37], [38] (see Detrended Fluctuation Analysis) and found no significant long-term memory for *w^1118^* either, p>0.6, **Figure S4**. In contrast, tethered flight activity has been shown to have long-range memory [21]. We analyzed the data from [21] with the tools used here and, consistent with [21], found tethered flight to have significant short and long-term memory, especially under close-loop conditions (see “onestripe”, **Figure S4**).

Because the IAI distribution (as measured by burstiness *B*) and memory (as measured by memory coefficient *M*) are two completely different mechanisms for bursty dynamics, a more complete characterization of a system can be made in a *B*-*M* plot. In this 2-D representation we can compare *Drosophila* dynamics and previous results for human behavioral dynamics [8]. *Drosophila* IAI dynamics lie in the same region as human dynamics, **Figure 2C**. This region corresponds to bursty dynamics mainly due to burstiness *B* and weakly to memory *M*. This is in contrast to meteorological or earthquake bursty dynamics that have more important memory effects, or to the distances between consecutive occurrences of a given letter in a text, which display a very low degree of burstiness [8]. This makes the dynamics of *Drosophila*, like human dynamics, even harder to predict than earthquakes or meteorological phenomena.

### Mushroom body decision-making circuitry is implicated in burstiness

To explore the possible implication of decision-making circuitry on behavioral burstiness, we began by selectively disrupting mushroom body (MB) signaling by using the GAL4/UAS system [32] that allows the expression of a temperature-sensitive form of dynamin, *shibire* (*shi^ts1^*). At permissive temperatures (<29°C) the synapses work normally, but at restrictive temperatures (>29°C) synaptic functioning ceases within minutes [33], [39]. Burstiness was assessed in the line 247-GAL4/UAS-*shi^ts1^* (‘247’), as it was found to have impaired choice behavior in a visual salience-based assay where flies were confronted with contradictory cues [20]. We also tested four more MB lines: c309-GAL4/UAS-*shi^ts1^* (‘c309’), 201Y-GAL4/UAS-*shi^ts1^* (‘201Y’), 17d-GAL4/UAS-*shi^ts1^* (‘17d’) and H24-GAL4/UAS-*shi^ts1^* (‘H24’). Line c309 was found to spend more time active, 247 and 17d to have no significant change and 201Y and H24 to spend less time active, **Figure S5**. This also allowed us to use these lines to control that it is not general changes in activity that cause changes in burstiness. After an initial day of adaptation to the experimental set-up, flies were monitored for three days at 23°C (permissive temperature, PT) to obtain baseline values, and were then switched to 31°C (restrictive temperature, RT) for three additional days (although frequently only the first day of RT was used for analysis as many flies could not survive for several days at the higher temperature). Differential parameters were then calculated from the values at RT minus the values at PT for each fly, to properly compare the genotypes under heat treatment.

**Figure 3:**
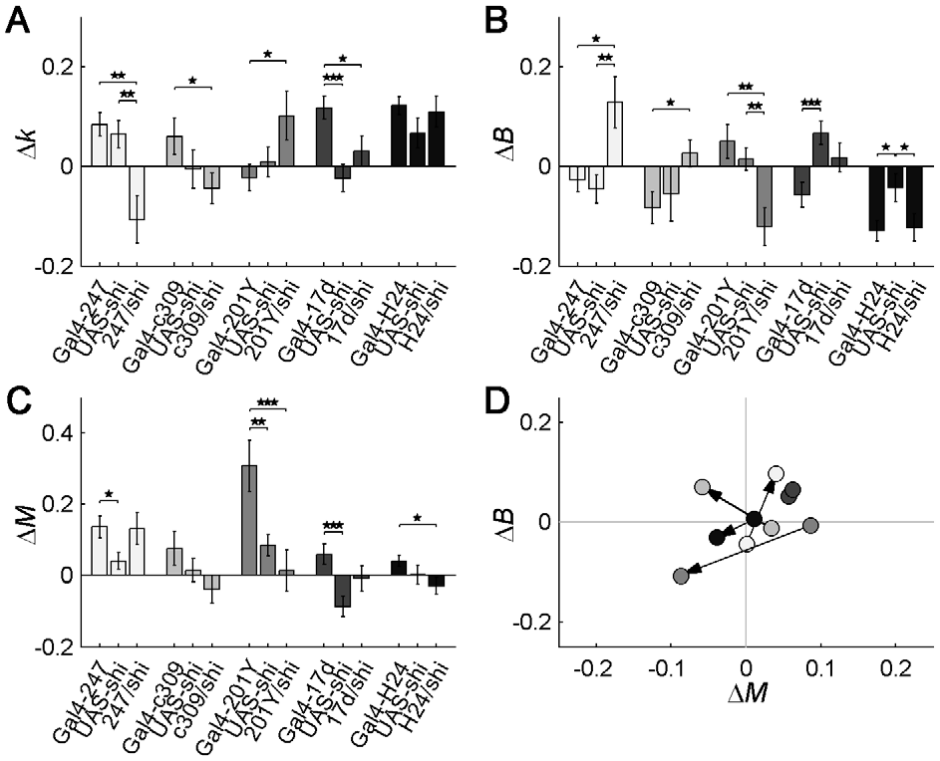
Impairment of mushroom body (MB) function affects burstiness. Panels (A–C) represent the change in parameter (*k*, *B* or *M*) of each genotype, between the restrictive temperature (RT) and the permissive temperature (PT, baseline values), i.e., “Δ = RT - PT”. Blocking neurons with line 247/*shi* increased burstiness (A, B) and blocking neurons with line 201Y/*shi* decreased burstiness (B), while targeting c309/*shi*, 17d/*shi* or H24/*shi* neurons did not produce any significant changes (A, B). (C) None of the MB lines caused significant changes of the memory parameter. (D) Representation of the net effect of blocking driver-specific transmission in the MB, approximately discounting the heat effect. Here, the values (dots) are calculated as the Gal4/UAS-*shi* construct's value minus the mean value of the two controls (i.e., “Δ = Gal4/*shi* – mean(Controls)”). Base of arrow indicates PT and head of arrow indicates RT. Note how the differences in burstiness (Δ*B*) for all MB lines are close to zero at PT, which indicates that when the Gal4/UAS-*shi* constructs had normal MB function the values of *B* were similar to that of the controls. Number of flies n = 18–32, error bars represent mean ± s.e.m. Corresponding activity level, *k* and *B* for IAIs and activity bouts for the MB strains are shown in Figure S5.

Transgenic 247 flies showed a mean increase of burstiness of 16.9% (*k*) and 17.1% (*B*) at RT (p<0.004, **Figure 3A** and p<0.013 **Figure 3B**) as compared to controls, while no concomitant change in mean activity was observed (p>0.08, **Figures S5A** and **S5D**). Lines c309, 17d and H24 did not show a significant difference in burstiness compared to controls, while line 201Y showed a statistically significant decrease (10.9–14.8%) in the burstiness parameter *B* (p<0.005), **Figure 3B**. None of the MB *shibire^ts1^* lines showed any significant changes in the memory parameter *M*, **Figure 3C**. By subtracting the mean *B* and *M* of the control lines from the transgenic's *B* and *M* at PT and RT, we obtained the approximate net effect of silencing MB circuitry without the conditional heat-effect, summarized in the *B*-*M* plot, **Figure 3D**. Analyzing the effect of total activity level on burstiness, we found that changes in burstiness were not correlated with time spent in activity/inter-activity (**Figures S5A–S5C** and **S5D–S5F**).

To complete the study of behavioral timing, we also analyzed the activity bout durations (ABs), **Figures S5D–S5F**. Line H24 showed a significant increase in the burstiness parameter *B* applied to ABs (p<0.01) and a decrease of the shape parameter *k* applied to ABs (p<0.05). None of the other MB lines displayed any significant changes in the *k* and *B* parameters applied to ABs (p>0.05 in **Figure S5F**).

Summarizing the MB disruption experiments, we found that the line 247 that was implicated in decision-making [20] also affected burstiness, as well as 201Y which affected burstiness in the opposite direction than 247. The other MB function-deficient lines c309, 17d and H24 did not change the internal fine structure of the IAIs.

### Dopamine levels affect burstiness

**Figure 4:**
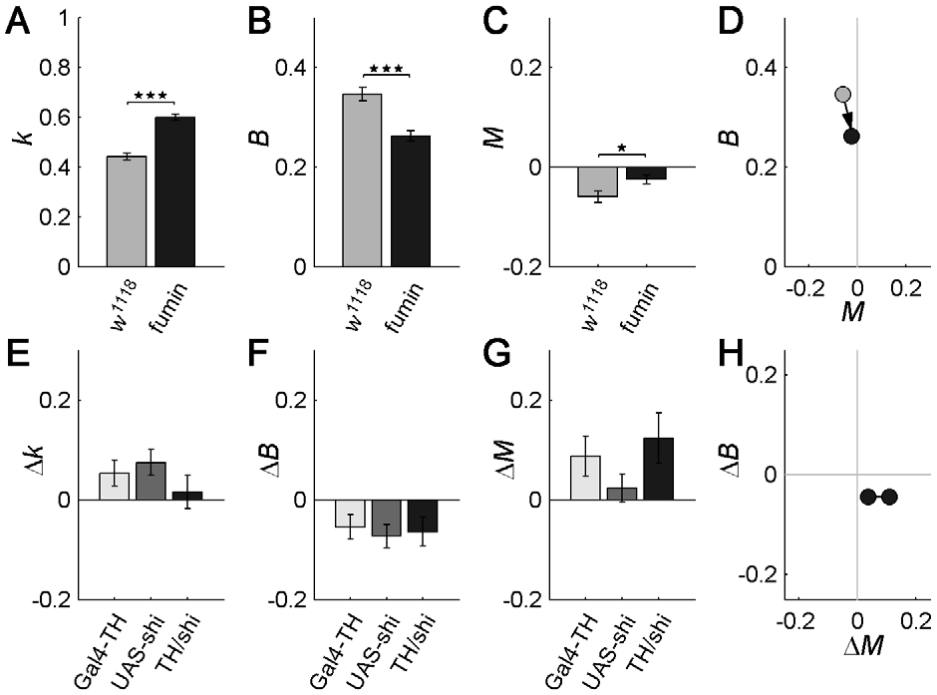
Behavior becomes more random with increased dopaminergic signaling, with lower burstiness and no memory effects. (A–D) Effect of increased dopamine (DA) levels in *fumin*, compared with control line *w^1118^*. A reduction in burstiness is seen as an increase of the shape parameter *k* (A) or as a decrease of the burstiness parameter *B* (B). This indicates that the activity pattern of *fumin* (high DA) displays less structure (is more Poissonian/random) than that of control flies (normal DA). (C) Both control *w^1118^* and *fumin* hardly display any memory effects *M* in the time series of IAIs, which means that the change in burstiness observed in *fumin* originates in a shift of the IAI distribution. (D) Burstiness and memory for *w^1118^* and *fumin*, shown in *B*-*M* plot. Base of arrow indicates control strain *w^1118^*, while head of arrow indicates *fumin*. (E–H) Disruption of dopaminergic signaling in TH/*shi* flies during restrictive temperature does not produce any significant change in burstiness (E, F) or memory (G), compared with controls. Differential values represent the change in parameter (*k*, *B* or *M*) of each genotype, during the restrictive temperature (RT) as compared with permissive temperature (PT, baseline values), i.e., “Δ = RT - PT”. (H) Net effect of silencing dopaminergic neurons, approximately discounting the heat effect. Here, the differential values represent the difference between the TH/*shi* line and the mean of the two control lines (i.e., “Δ = Gal4/*shi* – mean(Controls)”) at PT (right dot) and RT (left dot). Number of flies n = 29–64, error bars represent mean ± s.e.m. In Figure S6 burstiness nominal values for both *fumin*, TH/*shi* and controls are shown, for both IAIs and ABs.

We next studied the implication of dopamine (DA) on burstiness in *Drosophila*, as it has also been found to disrupt normal decision-making [20], [24], [25]. To examine what role dopamine plays, we exploited the fact that dopamine signaling can be both enhanced and silenced in *Drosophila*. The *fumin* (*fmn*) mutant has a genetic lesion in the dopamine transporter gene, which results in increased dopamine in the synaptic cleft [40]. Increased dopamine levels resulted in a 38.0% increase of the shape parameter *k* (p<0.0001, **Figure 4A**), and a concomitant decrease of 22.6% in the burstiness parameter (p<0.0001, **Figure 4B**). No effect in the memory coefficient *M* was observed (p = 0.136, **Figure 4C**). These results are summarized in the *B-M* plot, with the mutant strain being closer to Poissonian behavior than the control strain, **Figure 4D**.

We also examined the effect of reducing dopaminergic signaling. To silence dopaminergic neurons we expressed *shibire^ts1^* with the TH-GAL4 driver, using the same permissive/restrictive temperature protocol as for the transgenic MB lines. Tyrosine hydroxylase (TH) is an enzyme necessary for the proper synthesis of dopamine and present in most dopaminergic neurons [41]. Silencing dopaminergic signaling, as opposed to increasing it with *fumin*, did not result in any change of shape, burstiness or memory parameters (p>0.168, **Figures 4E–4G**). The net change of the controls subtracted from the TH-GAL4/UAS-*shi^ts1^* is summarized in the *B*-*M* plot in **Figure 4H**. While a reduction of dopamine levels did not have any effect on the initiation of activity, that is, on IAI burstiness, it did affect the *B* parameter of the activity bout durations, increasing it by 10.6–9.3% (p<0.03, **Figures S6E–S6F**). As in the case of MB disruption, no clear correlation between total activity (**Figures S6A** and **S6C**) was found with either shape parameters (**Figures S6B** and **S6E**), burstiness parameters (**Figures S6C** and **S6F**), or memory parameters (**Figures S6A, 4C and 4G**). The study of dopamine signaling shows that the increase of dopamine levels makes animal behavior more random, while its decrease has an effect on the dynamics of activity bout maintenance.

### Impairment of central complex function does not affect burstiness

To complement the study of decision-making and burstiness, the ellipsoid-body (EB) of the central complex was further tested as it has been previously implicated in the formation of power-law distributions [28], [29], and because some of the MB driver lines show expression in the EB. In [20] line C507-GAL4/UAS-*shi^ts1^* (‘C507’), with expression in the EB [42], was found not to affect decision-making. Using the same experimental design as previously described, we found that it presents no change in burstiness or memory, **Figure S7**. Lines C819-GAL4/UAS-*shi^ts1^* (‘C819’) and C232-GAL4/UAS-*shi^ts1^* (‘C232’) with expression in EB ring neurons [29], [43], and 78Y-GAL4/UAS-*shi^ts1^* (‘78Y’) with wider CX expression [28] were also analyzed, and we found no significant changes in burstiness or memory for any of these lines, **Figure S7**.

## Discussion

We used *Drosophila melanogaster* as an ideal system to experimentally test the link between decision-making and behavioral bursts used in recent mathematical models [11], [12]. *Drosophila* burstiness was found to be well described by the Weibull distribution. Further, *Drosophila* dynamics were found to be similar to human dynamics in the values of the burstiness and memory parameters [8]. To assess the link between decision-making and burstiness, we applied two different measures of burstiness to fly lines known to have disrupted choice behavior [20]. Importantly, we found that disrupting decision-making circuits impacted the degree of burstiness, in accordance with the proposed link.

The strongest influence on burstiness was found to be increased dopaminergic signaling. Dopaminergic neurons innervate the MB heavily, especially the lobes, as well as the CX and several other neuropils [20], [41], [44], [45]. The MB was also found to affect burstiness. In particular, line 247 has been directly implicated in decision-making [20] and we have found it to be implicated in burstiness, **Figure 3A,B**. This line has strong expression in the MB, with some additional weak expression in the ellipsoid body of the CX [46]. The other MB line we have found to be implicated in burstiness is 201Y, **Figure 3A,B**. This line has strong expression in the MB and no expression in the CX [46], further supporting that correct functioning of MB is necessary for normal burstiness. We did not find a modification of burstiness in MB lines c309, 17d and H24, **Figure 3A,B**. Also, we did not find any significant effect on burstiness in line C507, expressing in the EB, **Figure S7A,B**, previously shown not to affect decision-making [20]. We further tested other EB/CX lines (C819, C232, and 78Y) and again found no significant changes in burstiness, **Figure S7A,B**. Previous work had observed a disruption of power law behavior in CX lines [28], [29]. Some differences in our approach include using *shibire* instead of tetanus toxin to have more temporal control, the use of the genetic background *w^1118^* instead of *Canton-S* and, importantly, that we applied our analysis tools to the stationary portion of the data and to the complete set of inter-activity intervals, which closely follow a Weibull distribution.

Interestingly, the MB lines 247 and 201Y have similar expression patterns [46]. They both have very strong expression in the MB α/β lobes and in the γ lobe, no expression in the α'/β' lobes and either no expression or weak expression in other parts of the brain. The other MB lines show different expression patterns [46]. Line c309 also has some expression in the α'/β' lobes and relevant expression in most of the brain. Line 17d has only strong expression in the α/β lobes and none in the γ lobe. Line H24 has strong expression in the γ lobe and very weak in the α/β lobes and also shows strong expression in other parts of the brain, including the CX. Our results are thus most consistent with an implication of the α/β lobes and γ lobe. Notably, lines 247 and 201Y have an interesting difference in the expression pattern in the α/β lobes while their expression in the γ lobe is very similar [46]. While 247 shows a stronger expression in the surface and posterior subdivision of the α/β lobes, 201Y has its stronger expression in the core of these lobes. We note that while these two lines show modifications in burstiness, 247 shows an increase and 201Y a decrease, suggesting different roles for core and surface regions of the α/β lobes.

Taking advantage of the vast community knowledge on *Drosophila*, we can further suggest a closer relationship between neuroanatomical structures and the proposed mathematical models. Functions known to depend on the MB α/β lobes are the retrieval, but not acquisition, of olfactory memories [47] and the regulation of habituation responses [48]. Dopaminergic neurons in turn, have been found to disrupt aversive olfactory memory retention in [49] and convey motivational state by modifying MB memory processing in an internal-state dependent manner [50]. In this study we found that when dopamine signaling was enhanced, the bursty locomotor behavior was decreased, that is, timings of activity became more random. This is consistent with a model where *decision-making* is the result of weighing different sensory impulses with motivational states and memories of past outcomes through the interplay of the MB and DA systems. In a priority list task-execution model [11], [12], this decision-making process assigns priorities to the different impulses or options of attention or action. Future developments in the quantitative study of decision-making and its relation to burstiness will allow for a more detailed mechanistic description, but the fundamental link as proposed by the Barabási model is here shown to apply. In particular, when the dopaminergic system is hyper-excited or the function of the α/β and γ lobes is impaired, the balance or relative importance of different behavioral options breaks down, disrupting the decision-making processes and the proper establishment of priorities. Work in priority list models has shown that burstiness follows from priority lists with as few as two items, and that the outcome is independent of the specific function of priority assignment [11]. Hence, when an animal is repeatedly faced with two options or more, and chooses to first execute the most highly prioritized (e.g. by salience or other processes), the behavior becomes bursty, while if the animal acts on impulses as they come the behavior becomes more random, resembling what we have seen with the over-stimulated dopaminergic signaling.

The co-localization of decision-making and control of burstiness is thus consistent with the proposed mathematical model [11], [12], where a priority-driven base of action gives rise to the observed burstiness. We hope that with the rapid advancements in precise neural targeting, where small clusters or even single neurons can be identified and modified, the decision-making circuitry can be addressed with increasingly greater detail. This could provide the basis for more detailed and specific models of priority-driven decision-making processes, based on anatomical and functional knowledge of the circuitry. We also foresee that such models could further the understanding of the algorithms used by animals to produce optimal search behavior, without prior knowledge of the location of the resources [1], [7], [9], [34], [35]. Moreover, we foresee that the burstiness analysis described here could prove to become a useful tool for probing such neural circuitry, and aid in the finding of decision-making components.

## Materials and Methods

### Fly strains and rearing

Common genetic background strains *Canton-S*, *w^1118^* and *yellow-white* were kindly provided by I. Canal and J.F. Celis (U. Autónoma de Madrid and Centro de Biología Molecular, Spain), while *fumin* was kindly provided by K. Kume (U. Kumamoto, Japan). MB driver c309-GAL4 was obtained from the Bloomington *Drosophila* Stock Center, while lines 247-GAL4, 201Y-GAL4, 17d-GAL4, H24-GAL4, C507-GAL4, C819-GAL4, C232-GAL4, TH-GAL4 and UAS-*shi^ts1^* were kindly provided by A. Ferrús (Instituto Cajal, Spain) and line 78Y-GAL4 by J.R. Martin (CNRS, U. Paris-Sud). Heterozygote lines of Gal4 and UAS on a *w^1118^* background were used throughout. Stocks were maintained at 18°C on a standard cornmeal food, on a 12 h light/12 h dark cycle starting at 8:00 AM.

### Activity assay

Locomotion data were obtained with the DAM2 System (Trikinetics, Waltham, MA), which is a detector system with infra-red beams that cross through the center of 32 tubes of 65 mm length and 5.5 mm inner diameter. The flies are placed in the tubes individually, and the tubes are sealed with enough food for the duration of the experiment in one end and with a cotton plug in the other. When a fly crosses the beam an activity event is registered for that fly. Data were collected in 1 minute bins. It is known from observations and video-recordings that when flies are active they walk from one end of the tube to the other, usually without turning back before reaching the end of the tube, such that a minute with a registered activity event can truly be considered ‘active’, and that during inactive time periods the flies are in a rest behavior adopting a supported position, and are either completely immobile or performing some twitches of extremities, proboscis and abdomen [3],[51]. The experiments were performed inside incubators at 23°C (unless otherwise indicated), with no external stimuli, apart from the light cycle. Both male and virgin female flies were used for the experiments, and were 3–7 days old at the start of the experiment, unless otherwise noted.

### Burstiness analysis

Activity data were analyzed in Matlab R2007b (The MathWorks, Inc., MA) with a home-written analysis program, that can be downloaded from http://www.neural-circuits.org/flysiesta. Recordings were divided into activity bouts (ABs) and inter-activity intervals (IAIs). The survival distributions were constructed and fitted to the corresponding survival Weibull distribution exp(−(*x*/*λ*)*^k^*). A robust fit was found plotting log(−log *y*) against the variable *x*′ = log(*x*), for which the cumulative Weibull reduces to a line of the form *k⋅x*′+*C*, with *C* = −*k*⋅log(*λ*). To assess the quality of the fitting method for our type of data, we created artificial data sets from Weibull distributions with known *k* and *λ*, and also a variable number of data points to test the sample size dependence, **Figure S2**. By comparing the underlying parameter values with the ones obtained by different fitting techniques, we found that the linear fitting method is the most accurate in finding the underlying values, with a mean correlation coefficient of 0.9994 (p = 1.5e-08) in the range of *λ* = 5–25; *k* = 0.2–1.4 (with n = 30 to simulate the number of flies and 50–250 data points, which is typically the number of IAIs a fly has in the dark period).

### Detrended Fluctuation Analysis (DFA)

We tested for long-term memory using detrended fluctuation analysis [37], [38]. We compared the actual data against shuffled versions to calculate the significance level of long-term memory. A Matlab-based routine was written for this purpose, downloadable from http://www.neural-circuits.org/other-software.

### Statistical analysis

Statistical analysis was performed in Matlab with two-tailed Student's t-test, using the Bonferroni correction when conducting multiple comparisons. In cases where data met requirements of normality, tested with a Lillie-test, the parametric t-test was used. If requirements were not met, hypothesis testing was performed by bootstrapping the t-statistic (sampling with replacement and computing the t-statistic), using 10.000–100.000 sampling iterations. All error bars represent the standard error of the mean (s.e.m.), unless otherwise noted. In all figures the p-value of the statistical test is represented as either one star (p<0.05), two stars (p<0.01) or three stars (p<0.001).

## Supporting Information

Figure S1 **Activity patterns for three standard genetic background lines, and log-log representation of survival distribution data.** (A) For each animal, we measured the locomotor activity for 3 days and calculated the average daily pattern. Here we plot the mean daily pattern of the population. ZT = 0 denotes the start of the subjective day (lights on, white background) and ZT = 12 the start of the subjective night (lights off, grey background). Data are from 3-day-old flies from Figures 1 and 2. Blue line: *Canton-S*, green line: *yellow-white*, red line: *w^1118^*. (B) Log-log plot of the same IAI survival data as in Figure 1A (black error bars) and the Weibull fit (grey line). Red line is a power law fit (exponent = −0.525, r^2^ = 0.996) to IAI durations of 1–17 minutes – time interval approximately corresponding to the 1–1000 seconds used in ([4], Figure 9). Although a straight region can be found, for longer IAIs the distribution diverges considerably from a power law. The Weibull distribution (grey line) fits the data well for all IAI durations. In both panels, error bars represent the standard error of the mean (s.e.m.).
(TIF)

Figure S2 **The fit method correctly estimates the underlying parameters** ***k*** **and** ***λ*** **of the Weibull distribution.** To test that the fitting technique used to obtain the parameters *k* and *λ* for real fly data is accurate, we performed two different kinds of fits (‘Linear’ and ‘Non-linear’) to artificial data with known parameters. 50 (red), 100 (orange) 150 (green) 200 (light blue) or 250 (dark blue) points were randomly drawn from a Weibull distribution, with parameters in the ranges *k* = 0.2–1.4 and *λ* = 5–25. The randomly drawn values were then discretized in bins of 1, to mimic the real DAM System fly data, and the survival distribution was constructed. The Weibull survival distribution is given by *y* = exp(−(*x*/*λ*)*^k^*), and the Non-linear fit was obtained by fitting log(*y*) = −(*x*/*λ*)*^k^* with Matlab R2007b Curve Fitting Toolbox (“NonlinearLeastSquares” method), while the Linear fit was obtained by calculating the least squares regression of log(−log(*y*)) = *k⋅x*′+*C*, with *x*′ = log(*x*) and *C* = −*k*⋅log(*λ*). For each set of parameter values (*k*, *λ*) the procedure was repeated 30 times, to simulate the typical number of flies of each genotype. All error bars denote the standard deviation (s.d.) over the 30 independent runs. Accuracy of the fitting method to estimate *k* = [0.2∶0.1∶1.4], with *λ* = 15 in (A, B, E–G), and *λ* = [5∶5∶25], with *k* = 0.8 in (C, D, H–J). (A–D) Difference between the estimated parameter and the parameter of the underlying Weibull distribution the data was drawn from. The Linear fit is better at extracting both parameters, as it has less error and smaller standard deviations for all sample sizes. (F, I) Calculation of the sum of squared errors of the (random sample) survival distribution, to the real (parent) Weibull distribution the data was drawn from. For small *k*'s (*k*<0.5) and small sample sizes, R^2^ is relatively low (R^2^<0.9), but note that the underlying parameters are still correctly obtained (A, C). (E, G, H, J) Difference between the R^2^ obtained by least square fitting and the real R^2^. Even though the Non-linear fitting seems to do a ‘better’ fit because R^2^ is higher, the Linear fit is actually better at extracting the true parameters.
(TIF)

Figure S3 **Overview of three standard genetic background lines' activity and burstiness, at two different ages.** Flies of three commonly used genotypes (*Canton-S* (*CS*), *yellow-white* (*yw*) and *w^1118^*) were tested for activity and burstiness, both as young (3 days) and as adults (4 weeks), and found to display bursty dynamics, both for inter-activity intervals (IAI) and activity bout (AB) dynamics. (A) Total time spent in IAI in dark period (12 h), per day. (B, C) IAI burstiness measured with the shape parameter *k* or burstiness parameter *B*. (D) Total time spent active in the dark period, per day (complementary to total time in IAI). (E, F) Parameters *k* and *B* applied to AB dynamics. Data are the same as used for Figures 1 and 2, and represented as mean ± s.e.m.
(TIF)

Figure S4 **Short and long-term memory.** Flies of three commonly used genotypes (*Canton-S* (*CS*), *yellow-white* (*yw*) and *w^1118^*) and of two ages (3 days and 4 weeks) and tethered flight data from reference [21] were tested for (A) short-term and (B) long-term memory. Significance levels are computed by comparison of actual and shuffled data (white bars). Note that the genetic background used in this study, *w^1118^*, displays no significant memory. Contrast this, for example, with data from reference [21] of *WT Berlin* flies in tethered flight in closed-loop response to a stimulus stripe (‘onestripe’).
(TIF)

Figure S5 **Differential effect of mushroom body (MB) mutants on activity levels and burstiness.** Data of inter-activity intervals (IAI) and activity bouts (AB) for the MB-*shibire^ts1^* lines in Figure 3. Bars represent the change in the parameter value between permissive and restrictive temperatures (RT-PT); error bars indicate s.e.m. (A, D) Change of the total time spent in IAI (A) and AB (D) in dark period, per day. Blocking c309 neuronal function with *shibire* causes the flies to become significantly hyperactive, while blocking 201Y or H24 function renders flies less active than the controls. Silencing neurons targeted by lines 247 or 17d produces no significant change compared with controls. (B, C) Change in burstiness parameters *k* and *B*. Line 247 becomes significantly more bursty than controls, measured by both *k* and *B*, while line 201Y is less bursty than controls, statistically significant only with burstiness parameter *B*. No statistically significant change in burstiness occurs for c309, 17d or H24. (E, F) Change in parameters *k* and *B*, as applied to ABs. Silencing line H24 neurons causes a significant change in the AB maintenance dynamics, measured both with *k* and *B*, while the other MB lines produce no change in AB dynamics. Comparing the changes in activity level with the changes in burstiness, it can be concluded that burstiness does not correlate with general activity level.
(TIF)

Figure S6 **Effect of dopamine (DA) levels on activity and burstiness.** Nominal values for *fumin* (high DA levels) and TH/*shi* (normal DA levels at PT, low/null DA levels at RT), and their corresponding controls. (A, D) Total time spent in IAI (A) and AB (D) in dark period, averaged per day. High DA produces hyperactivity, while low DA causes inactivity. (B, C) High DA levels decrease the degree of behavioral burstiness, while lowering DA levels has no effect, seen as a significant change of *k* and *B* for *fumin*, but not for TH at RT with respect to PT. (E, F) Opposite action of DA level on AB maintenance dynamics: *fumin* lowers the internal structure of AB durations, while TH at RT significantly increases it. Data correspond to Figure 4 of the main text, bars indicate mean ± s.e.m.
(TIF)

Figure S7 **Impairment of central complex (CX) function does not affect burstiness.** Panels (A–C) represent the change in parameter (*k*, *B* or *M*) of each genotype, between the restrictive temperature (RT) and the permissive temperature (PT, baseline values), i.e., “Δ = RT - PT”. None of the CX lines, C507, C819, C232 and 78Y, caused significant changes of burstiness (A,B) or the memory parameter (C). (D) Representation of the net effect of blocking driver-specific transmission in the CX, approximately discounting the heat effect. Here, the values (dots) are calculated as the Gal4/UAS-*shi* construct's value minus the mean value of the two controls (i.e., “Δ = Gal4/*shi* – mean(Controls)”). Base of arrow indicates PT and head of arrow indicates RT. Note how the differences in burstiness (Δ*B*) are close to zero at PT, which indicates that when the Gal4/UAS-*shi* constructs have normal CX function the values of *B* are similar to that of the controls. Also note, if comparing with MB values (Figure 3), that the scale of the axes are different. Number of flies n = 25–30, error bars represent mean ± s.e.m.
(TIF)

Text S1 Supporting Material and Methods, regarding the Weibull parameters' relation to the mean IAI and to the burstiness parameter *B*.
(PDF)
