# Supplementary figures and images for: The Origin of Behavioral Bursts in Decision-Making Circuitry
Source: PLoS Comput Biol. 2011 Jun 23;7(6):e1002075. doi: 10.1371/journal.pcbi.1002075 (PMC3121695; doi:10.1371/journal.pcbi.1002075)

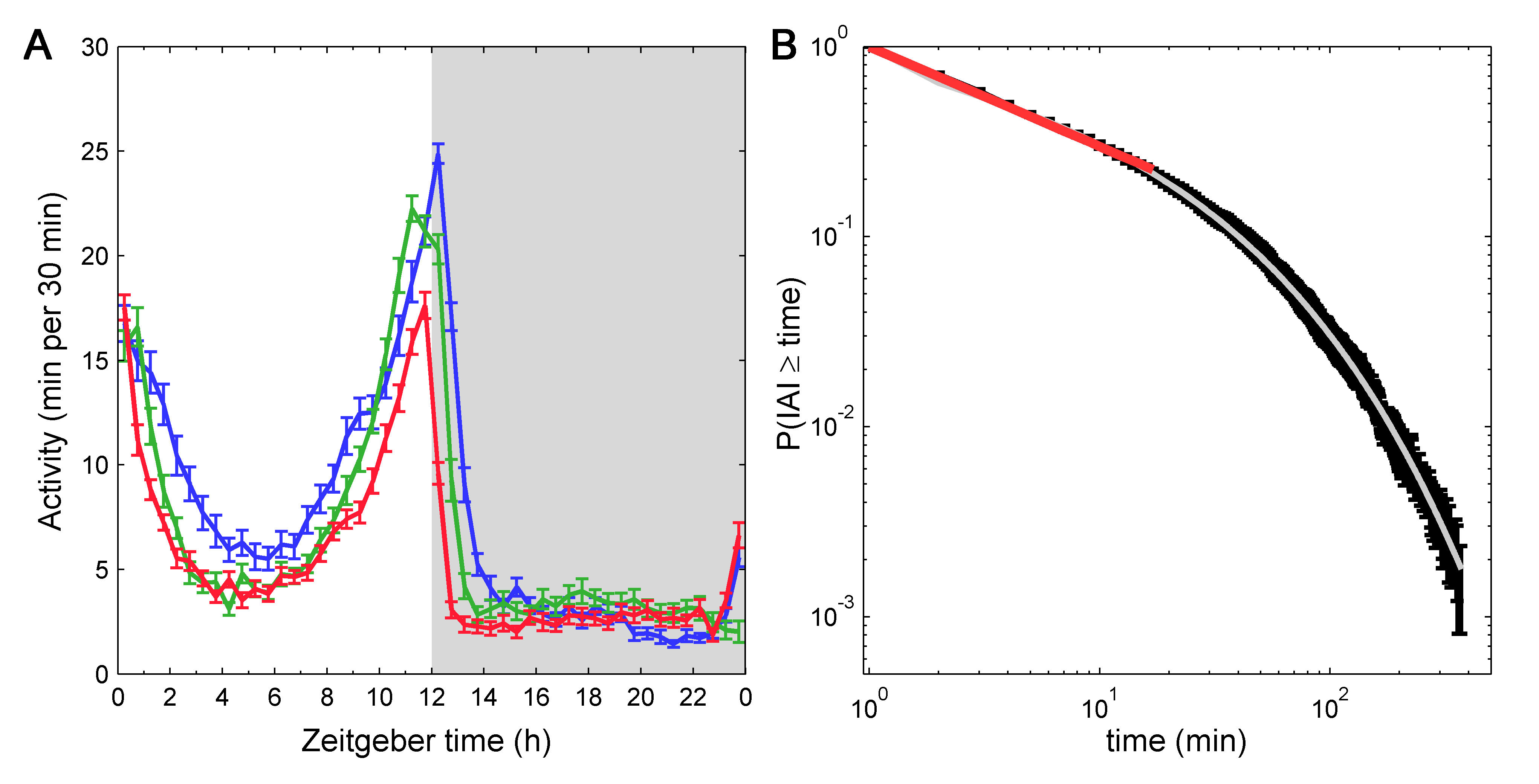

Supplement: Figure S1 — Activity patterns for three standard genetic background lines, and log-log representation of survival distribution data. (A) For each animal, we measured the locomotor activity for 3 days and calculated the average daily pattern. Here we plot the mean daily pattern of the population. ZT = 0 denotes the start of the subjective day (lights on, white background) and ZT = 12 the start of the subjective night (lights off, grey background). Data are from 3-day-old flies from Figures 1 and 2. Blue line: Canton-S, green line: yellow-white, red line: w1118. (B) Log-log plot of the same IAI survival data as in Figure 1A (black error bars) and the Weibull fit (grey line). Red line is a power law fit (exponent = −0.525, r2 = 0.996) to IAI durations of 1–17 minutes – time interval approximately corresponding to the 1–1000 seconds used in ([4], Figure 9). Although a straight region can be found, for longer IAIs the distribution diverges considerably from a power law. The Weibull distribution (grey line) fits the data well for all IAI durations. In both panels, error bars represent the standard error of the mean (s.e.m.). (TIF) [file pcbi.1002075.s001.tif]

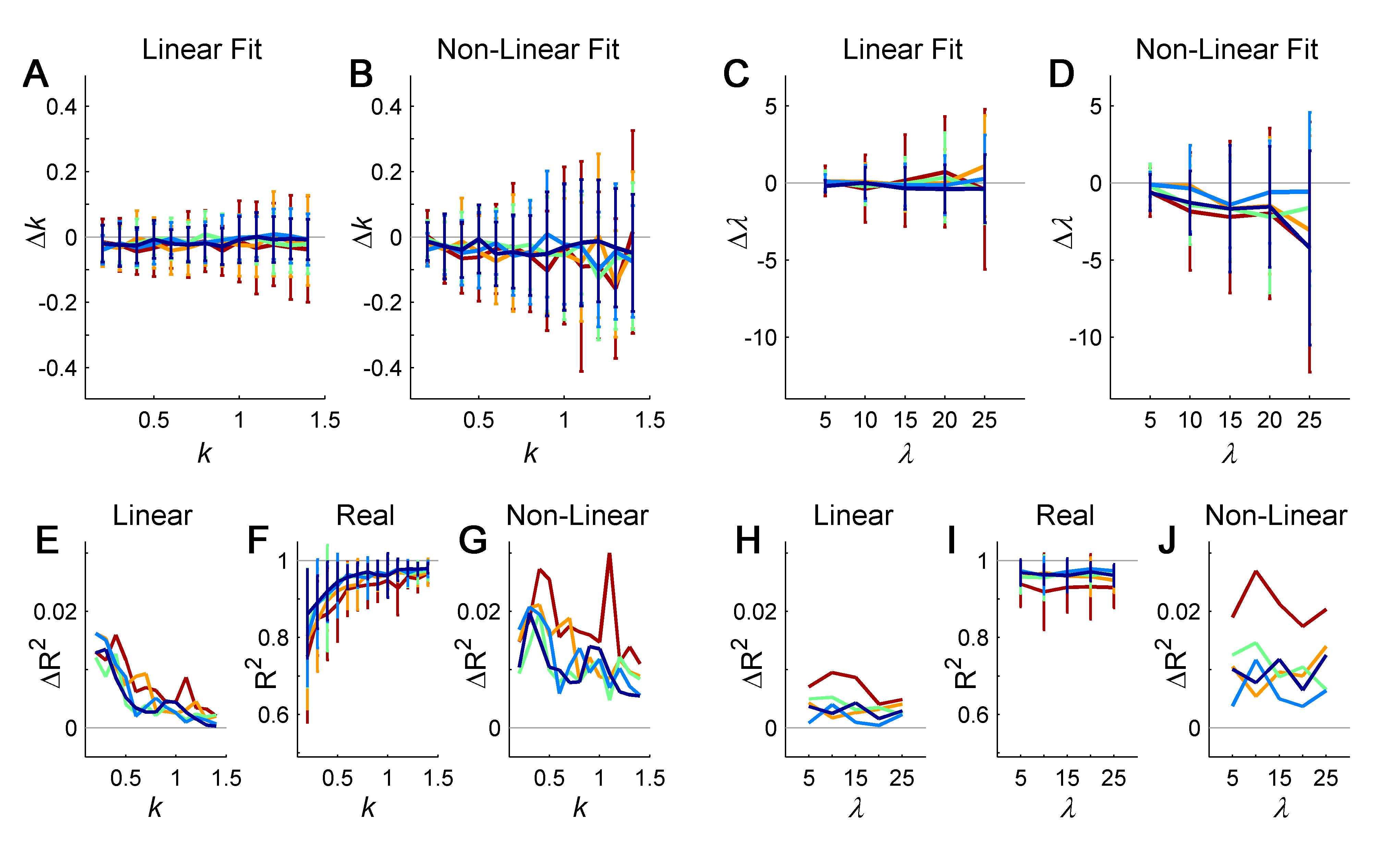

Supplement: Figure S2 — The fit method correctly estimates the underlying parameters k and λ of the Weibull distribution. To test that the fitting technique used to obtain the parameters k and λ for real fly data is accurate, we performed two different kinds of fits (‘Linear’ and ‘Non-linear’) to artificial data with known parameters. 50 (red), 100 (orange) 150 (green) 200 (light blue) or 250 (dark blue) points were randomly drawn from a Weibull distribution, with parameters in the ranges k = 0.2–1.4 and λ = 5–25. The randomly drawn values were then discretized in bins of 1, to mimic the real DAM System fly data, and the survival distribution was constructed. The Weibull survival distribution is given by y = exp(−(x/λ)k), and the Non-linear fit was obtained by fitting log(y) = −(x/λ)k with Matlab R2007b Curve Fitting Toolbox (“NonlinearLeastSquares” method), while the Linear fit was obtained by calculating the least squares regression of log(−log(y)) = k⋅x′+C, with x′ = log(x) and C = −k⋅log(λ). For each set of parameter values (k, λ) the procedure was repeated 30 times, to simulate the typical number of flies of each genotype. All error bars denote the standard deviation (s.d.) over the 30 independent runs. Accuracy of the fitting method to estimate k = [0.2∶0.1∶1.4], with λ = 15 in (A, B, E–G), and λ = [5∶5∶25], with k = 0.8 in (C, D, H–J). (A–D) Difference between the estimated parameter and the parameter of the underlying Weibull distribution the data was drawn from. The Linear fit is better at extracting both parameters, as it has less error and smaller standard deviations for all sample sizes. (F, I) Calculation of the sum of squared errors of the (random sample) survival distribution, to the real (parent) Weibull distribution the data was drawn from. For small k's (k<0.5) and small sample sizes, R2 is relatively low (R2<0.9), but note that the underlying parameters are still correctly obtained (A, C). (E, G, H, J) Difference between the R2 obtained by least square fitting and the re [file pcbi.1002075.s002.tif]

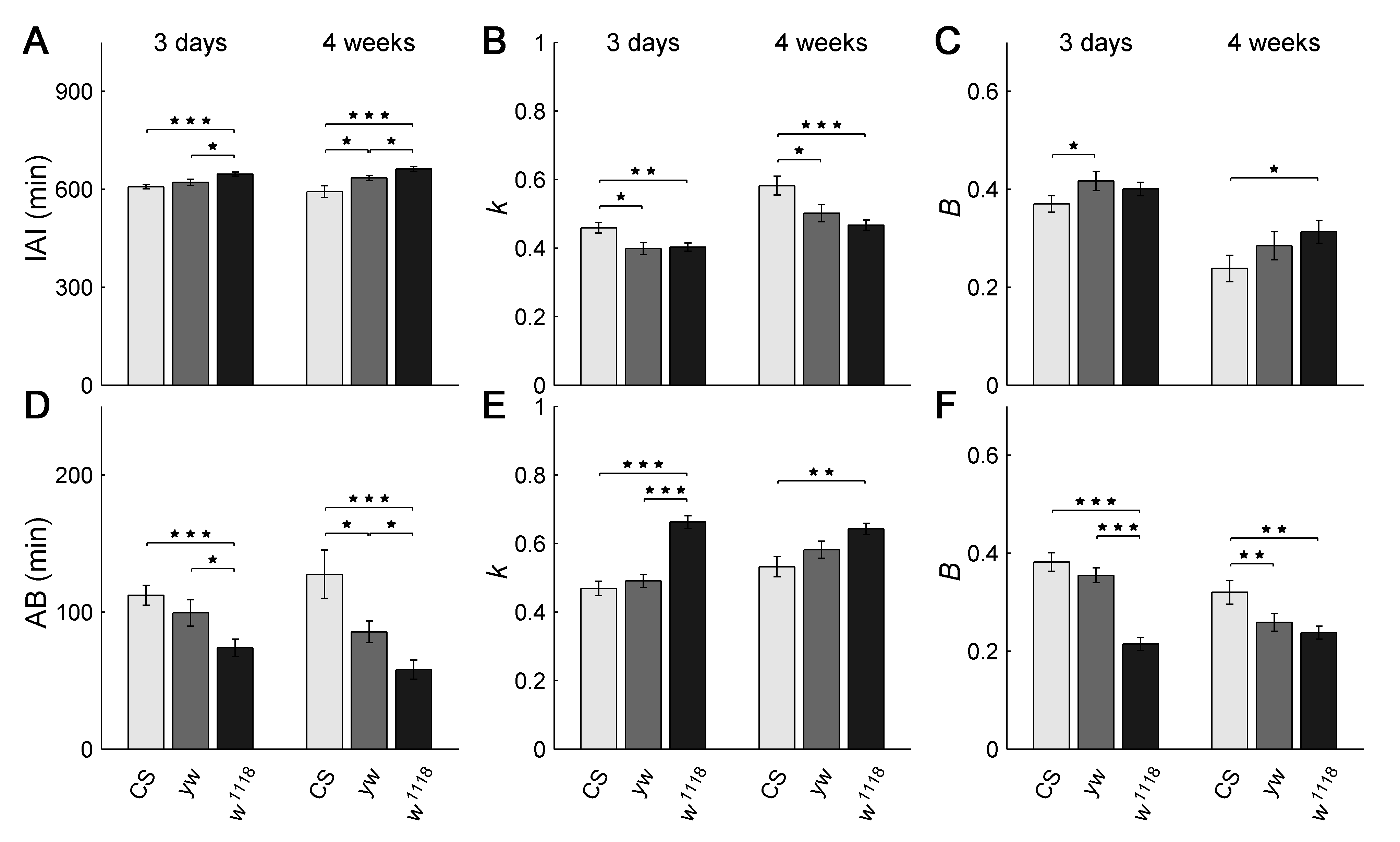

Supplement: Figure S3 — Overview of three standard genetic background lines' activity and burstiness, at two different ages. Flies of three commonly used genotypes (Canton-S (CS), yellow-white (yw) and w1118) were tested for activity and burstiness, both as young (3 days) and as adults (4 weeks), and found to display bursty dynamics, both for inter-activity intervals (IAI) and activity bout (AB) dynamics. (A) Total time spent in IAI in dark period (12 h), per day. (B, C) IAI burstiness measured with the shape parameter k or burstiness parameter B. (D) Total time spent active in the dark period, per day (complementary to total time in IAI). (E, F) Parameters k and B applied to AB dynamics. Data are the same as used for Figures 1 and 2, and represented as mean ± s.e.m. (TIF) [file pcbi.1002075.s003.tif]

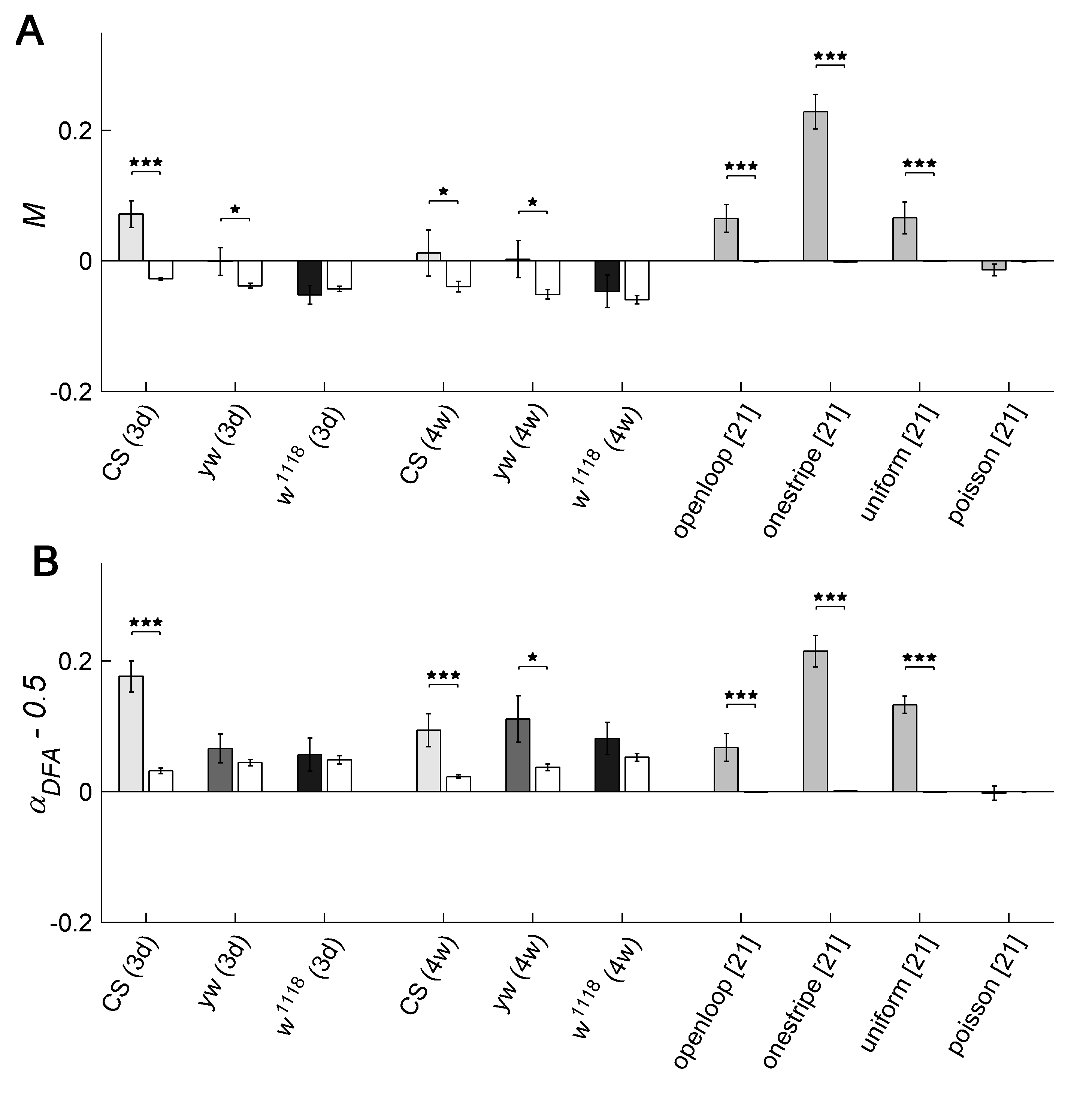

Supplement: Figure S4 — Short and long-term memory. Flies of three commonly used genotypes (Canton-S (CS), yellow-white (yw) and w1118) and of two ages (3 days and 4 weeks) and tethered flight data from reference [21] were tested for (A) short-term and (B) long-term memory. Significance levels are computed by comparison of actual and shuffled data (white bars). Note that the genetic background used in this study, w1118, displays no significant memory. Contrast this, for example, with data from reference [21] of WT Berlin flies in tethered flight in closed-loop response to a stimulus stripe (‘onestripe’). (TIF) [file pcbi.1002075.s004.tif]

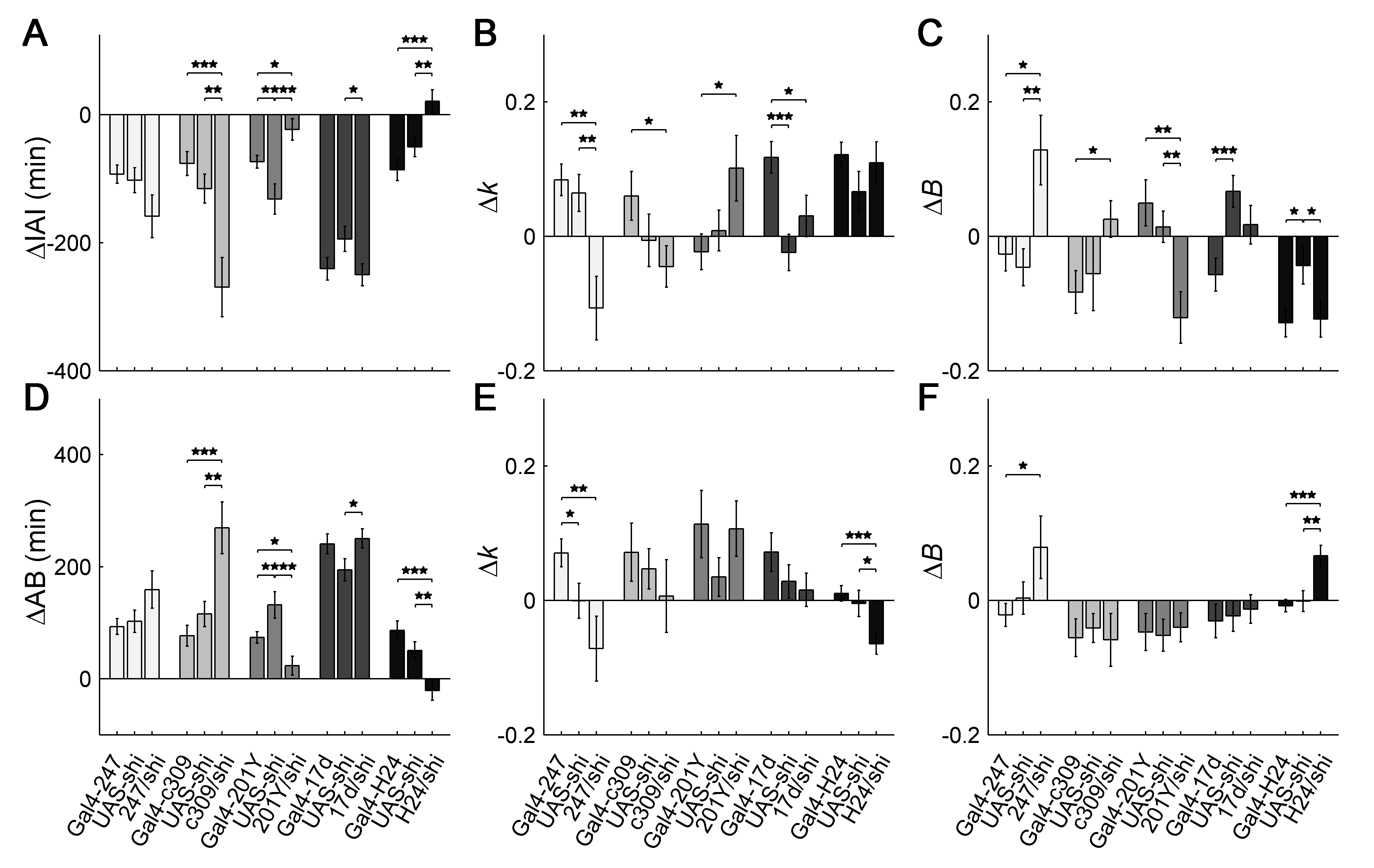

Supplement: Figure S5 — Differential effect of mushroom body (MB) mutants on activity levels and burstiness. Data of inter-activity intervals (IAI) and activity bouts (AB) for the MB-shibirets1 lines in Figure 3. Bars represent the change in the parameter value between permissive and restrictive temperatures (RT-PT); error bars indicate s.e.m. (A, D) Change of the total time spent in IAI (A) and AB (D) in dark period, per day. Blocking c309 neuronal function with shibire causes the flies to become significantly hyperactive, while blocking 201Y or H24 function renders flies less active than the controls. Silencing neurons targeted by lines 247 or 17d produces no significant change compared with controls. (B, C) Change in burstiness parameters k and B. Line 247 becomes significantly more bursty than controls, measured by both k and B, while line 201Y is less bursty than controls, statistically significant only with burstiness parameter B. No statistically significant change in burstiness occurs for c309, 17d or H24. (E, F) Change in parameters k and B, as applied to ABs. Silencing line H24 neurons causes a significant change in the AB maintenance dynamics, measured both with k and B, while the other MB lines produce no change in AB dynamics. Comparing the changes in activity level with the changes in burstiness, it can be concluded that burstiness does not correlate with general activity level. (TIF) [file pcbi.1002075.s005.tif]

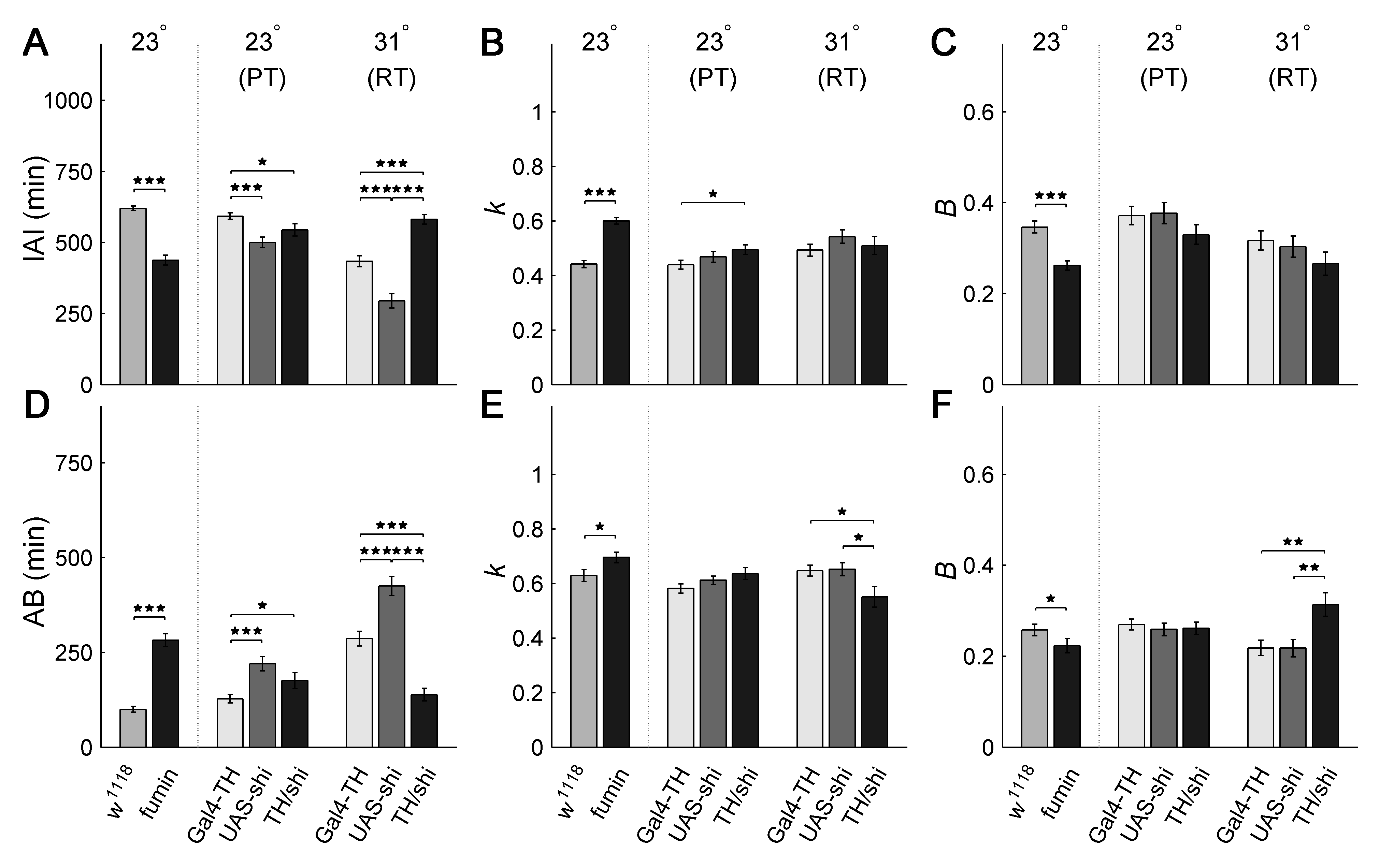

Supplement: Figure S6 — Effect of dopamine (DA) levels on activity and burstiness. Nominal values for fumin (high DA levels) and TH/shi (normal DA levels at PT, low/null DA levels at RT), and their corresponding controls. (A, D) Total time spent in IAI (A) and AB (D) in dark period, averaged per day. High DA produces hyperactivity, while low DA causes inactivity. (B, C) High DA levels decrease the degree of behavioral burstiness, while lowering DA levels has no effect, seen as a significant change of k and B for fumin, but not for TH at RT with respect to PT. (E, F) Opposite action of DA level on AB maintenance dynamics: fumin lowers the internal structure of AB durations, while TH at RT significantly increases it. Data correspond to Figure 4 of the main text, bars indicate mean ± s.e.m. (TIF) [file pcbi.1002075.s006.tif]

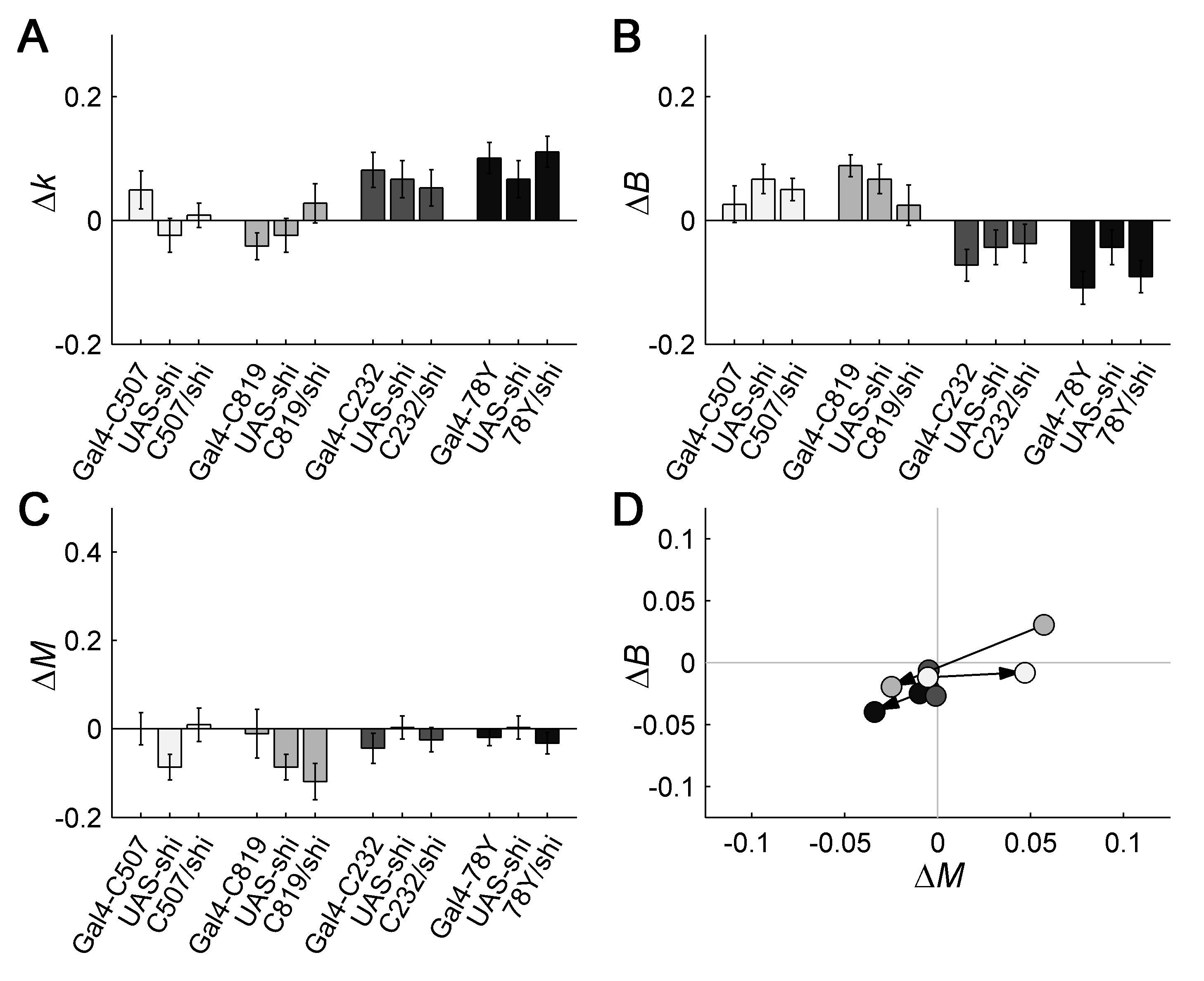

Supplement: Figure S7 — Impairment of central complex (CX) function does not affect burstiness. Panels (A–C) represent the change in parameter (k, B or M) of each genotype, between the restrictive temperature (RT) and the permissive temperature (PT, baseline values), i.e., “Δ = RT - PT”. None of the CX lines, C507, C819, C232 and 78Y, caused significant changes of burstiness (A,B) or the memory parameter (C). (D) Representation of the net effect of blocking driver-specific transmission in the CX, approximately discounting the heat effect. Here, the values (dots) are calculated as the Gal4/UAS-shi construct's value minus the mean value of the two controls (i.e., “Δ = Gal4/shi – mean(Controls)”). Base of arrow indicates PT and head of arrow indicates RT. Note how the differences in burstiness (ΔB) are close to zero at PT, which indicates that when the Gal4/UAS-shi constructs have normal CX function the values of B are similar to that of the controls. Also note, if comparing with MB values (Figure 3), that the scale of the axes are different. Number of flies n = 25–30, error bars represent mean ± s.e.m. (TIF) [file pcbi.1002075.s007.tif]
