# Supplementary material for: The Origin of Behavioral Bursts in Decision-Making Circuitry
Source: PLoS Comput Biol. 2011 Jun 23;7(6):e1002075. doi: 10.1371/journal.pcbi.1002075 (PMC3121695; doi:10.1371/journal.pcbi.1002075)
Supplement: Text S1 — Supporting Material and Methods, regarding the Weibull parameters' relation to the mean IAI and to the burstiness parameter B. (PDF) [file pcbi.1002075.s008.pdf]

Supporting Information for
The Origin of Behavioral Bursts in Decision-Making Circuitry

Supporting Material and Methods

(a) The mean IAI is directly proportional to the scale parameter *λ*

The *n*-th moment of the Weibull distribution is of the form [1]

$$\lambda^n \Gamma(1+n/k)\,. \quad \text{(S1)}$$

Thus the mean is given by

$$m=\lambda\Gamma(1+1/k)\,, \quad \text{(S2)}$$

that is linearly proportional to the parameter λ, as we wanted to show.
The dependence with the shape parameter k is through the gamma function. To get insight on this dependence consider the following approximation in the interval $0.2<k<1, \Gamma(1+1/k)\approx 0.25\ Exp[1.0757/k]$, that is, the mean increases exponentially with the inverse of the shape parameter k in the region of k of experimental interest to us, $0.2<k<1$.

(b) The burstiness parameter *B*, when applied to the Weibull distribution, is independent of the scale parameter

Substituting Eq. (S1) for n=1,2 into Eq. (2), we obtain,

$$B=\frac{\left(\Gamma(1+2/k)-\Gamma^2(1+1/k)\right)^{1/2}-\Gamma(1+1/k)}{\left(\Gamma(1+2/k)-\Gamma^2(1+1/k)\right)^{1/2}+\Gamma(1+1/k)}\,, \quad \text{(S3)}$$

that only depends on the shape parameter k. To get some intuition, we note that for the interval of k of interest in this paper, $0.2<k<1$, burstiness can be roughly approximated by $B\approx 1.27-2.26k+1.02k^2$, that is a function that decreases monotonically with k.

Supporting References

1. Johnson NL, Kotz S, Balakrishnan N (1994) Continuous Univariate Distributions, Vol.1. Wiley Series in Probability and Statistics.
